# Serum vascular endothelial growth factors C and D as forecast tools for patients with gallbladder carcinoma

**DOI:** 10.1007/s13277-015-3316-3

**Published:** 2015-03-24

**Authors:** Min-Chao Liu, Lei Jiang, Hai-Jie Hong, Ze-Wu Meng, Qiang Du, Liang-Yi Zhou, Fei-Fei She, Yan-Ling Chen

**Affiliations:** 1 Department of Hepatobiliary Surgery, the Affiliated Union Hospital of Fujian Medical University, 29 Xinquan Road, Fuzhou, Fujian 350001 People’s Republic of China; 2 Key Laboratory of Ministry of Education for Gastrointestinal Cancer, Fujian Medical University, Fuzhou, Fujian 350108 People’s Republic of China; 3 Key Laboratory of Tumor Microbiology, School of Basic Medical Sciences, Fujian Medical University, 1 Xueyuan Road, Fuzhou, Fujian 350108 People’s Republic of China

**Keywords:** Gallbladder carcinoma, Serum VEGF-C, Serum VEGF-D, Lymph nodes metastases, Prognosis

## Abstract

Gallbladder carcinoma (GBC) is the most common cancer of the biliary tract. Lymph node metastasis (LNM) is the major diffusion route of GBC and is a prognosis factor. The aim of study was to assess the potential of the serum VEGF-C and VEGF-D (sVEGF-C/D) levels to predict the presence of LNM and the survival of GBC patients. The preoperative sVEGF-C/D levels of 31 patients with GBC, 10 patients with cholesterol polyps, and 10 healthy volunteers were measured by enzyme-linked immunoadsorbent assay (ELISA). The sVEGF-C/D levels of patients with GBC were significantly higher than those of people with healthy gallbladders (*p* < 0.001 and *p* = 0.001, respectively) and cholesterol polyp (*p* = 0.032 and *p* = 0.004, respectively). In GBC, the sVEGF-C levels were associated with LNM (*p* = 0.011), distant metastasis (*p* = 0.018), and stage (*p* = 0.045), but the sVEGF-D levels had a significant association with the tumor depth (*p* = 0.001), LNM (*p* = 0.001), distant metastasis (*p* = 0.047), and stage (*p* = 0.002). The sVEGF-C/D diagnostic values for the presence of GBC were sensitivity of 71.0 and 74.2 % and specificity of 80.0 and 85.0 %, respectively. With respect to the diagnosis of LNM, the diagnostic values of sVEGF-C/D were as follows: sensitivity 81.2 and 87.5 % and specificity 73.3 and 80.0 %, respectively. The mean survival time with high sVEGF-C was significantly shorter than that with low sVEGF-C (*p* < 0.001), which was also true for low sVEGF-D (*p* = 0.032). The preoperative sVEGF-C/D levels might be reliable biomarkers for the presence of disease and LNM in patients with GBC. The sVEGF-C/D levels may be prognosis factors that can predict a poor outcome for GBC patients.

## Introduction

Gallbladder carcinoma (GBC) is the sixth most common cancer of the gastrointestinal tract and is most common cancer of the biliary tract [1]. According to epidemiological investigations, the incidence rates are extraordinarily high in Asia and relatively high in Korea (13.7/100,000). The 5-year survival rate is only 5 %, and the overall mean survival time is 6 months [2]. Radical resection is the most effective and only potentially curative treatment [3, 4]. However, because there are few effective diagnostic measures and classical symptoms, most of patients with GBC are treated at late stages of the disease, resulting in a poor overall prognosis [5]. Lymph node metastasis (LNM) is the major diffusion route of GBC. It is not only an important component of tumor staging but also a prognostic factor for GBC. Moreover, LNM often occurs in the early stage of GBC. Therefore, it is particularly important to identify a method that can exactly assess the LNM of GBC before an operation.

Vascular endothelial growth factors C and D (VEGF-C/D) have been identified as the important numbers of VEGF family and are considered as the lymphangiogenic factors. VEGF-C/D induce the formation of lymphatic ducts and promote lymph node metastasis in combination with VEGFR-3 [6]. Many recent studies have observed tumor VEGF-C/D overexpression in many carcinomas, specifically those whose major diffusion route is LNM, such as gastric carcinoma [7] and colorectal cancer [8]. The studies have shown that tumor VEGF-C/D overexpression is correlated with the presence of carcinoma and LNM and may be related to the prognosis. Moreover, our group [9] has found that VEGF-C/D are involved in the lymphangiogenesis in GBC and induce LNM of the tumor. VEGF-C/D might be useful for evaluating LNM and the prognosis in GBC.

Recently, studies on the serum VEGF-C/D (sVEGF-C/D) levels have become popular. Wang et al. [10] reported that the serum VEGF-C level is related to LNM and the poor prognosis of patients with gastric cancer, which has a positive correlation with the tumor VEGF-C expression. Lai et al. [11] demonstrated that the serum VEGF-D level correlates with the presence of cervical lymph node metastases and might be a useful prognostic indicator in papillary thyroid carcinoma patients. Therefore, we posit that the serum VEGF-C/D levels correlate with the tumor expression of VEGF-C/D and may be used as tumor makers and prognostic factors for GBC patients.

To date, few studies have demonstrated whether the serum VEGF-C/D levels predict the presence of LNM and indicate the survival of GBC patients. Few effective biomarkers from the serum of patients with GBC have been used in the clinic, particularly as diagnosis tools for LNM. The aim of the study was to evaluate the potential for the serum VEGF-C/D levels to be used in predicting the presence of LNM and survival of GBC patients.

## Materials and methods

### Patients

Between November 2008 and February 2014, serum samples were collected from 31 patients with histopathologically proven GBC who were treated with surgery but not preoperative radiochemotherapy or transfusion in the Affiliated Union Hospital of Fujian Medical University. Among the 31 GBC patients with a median age of 63 years (range from 43 to 83 years), 17 had adenocarcinoma, 4 papillary carcinoma, 3 mucinous adenocarcinoma, 3 tubular adenocarcinoma, 2 squamous carcinoma, 1 adenosquamous carcinoma, and 1 neuroendocrine carcinoma. Sixteen patients had lymph node metastasis (LNM), and 8 had distant metastasis. All cases were staged clinically according to the American Joint Committee on Cancer (AJCC, 7th) [12]. The clinicopathological characteristics of GBC patients are summarized in Table 1. The 31 patients underwent follow-up for at least 5 months (range 5–62 months) via telephone.

**Table 1:** Association between sVEGF-C/D levels and clinicopathological feature of GBC

Parameters	Number	VEGF-C (pg/ml)^a^	*p* value	VEGF-D (pg/ml)^b^	*p* value
Age					
< 63 years	15	8041.35 ± 1925.64	0.327	703.88 (548.00–1465.13)	0.220
≥ 63 years	16	7321.04 ± 2089.88		660.38 (446.50–1599.25)	
Sex					
Female	16	7318.29 ± 1970.20	0.324	644.06 (461.00–1149.75)	0.105
Male	15	8044.30 ± 2055.27		776.38 (446.50–1599.25)	
Smoking status					
Smoker	12	8060.85 ± 2277.43	0.399	754.63 (446.50–1599.25)	0.543
Non-smoker	19	7422.46 ± 1856.82		656.75 (461.00–1149.75)	
Location					
Neck	7	7863.53 ± 2196.52	0.336	671.25 (548.00–1465.13)	0.082
Body	3	6775.27 ± 734.17		569.75 (558.88–645.88)	
Bottom	9	6884.40 ± 2161.90		602.38 (446.50–921.38)	
Whole	12	8368.91 ± 1905.67		796.31 (508.13–1599.25)	
Tumor size					
< 4 cm	14	7797.32 ± 2166.12	0.754	627.75 (446.50–1465.13)	0.341
≥ 4 cm	17	7564.38 ± 1936.99		703.88 (508.13–1599.25)	
Histological type					
Adeno	17	7480.00 ± 2219.24	0.572	671.25 (446.50–1599.25)	0.451
Others	14	7899.79 ± 1782.19		696.63 (453.75–1465.13)	
Histological grading					
Poor	11	8360.97 ± 1974.50	0.378	703.88 (529.88–1465.13)	0.792
Moderate	15	7302.74 ± 1955.52		671.25 (453.75–1599.25)	
Well	5	7249.04 ± 2278.15		689.38 (446.50–943.13)	
Tumor depth					
Tis-T2	10	6788.88 ± 1945.62	0.188	544.38 (446.50–758.25)	0.001*
T3	16	7906.59 ± 1756.33		799.94 (642.25–1465.13)	
T4	5	8672.56 ± 2618.21		678.50 (548.00–1599.25)	
LNM					
N0	15	6807.64 ± 1663.66	0.011*	602.38 (453.78–823.50)	0.001*
N1	9	7725.51 ± 2142.83		751.00 (446.50–1149.75)	
N2	7	9444.69 ± 1456.74		946.75 (678.50–1599.25)	
Distant metastasis					
M0	23	7177.17 ± 1767.94	0.018*	671.25 (446.50–1073.63)	0.047*
M1	8	9085.27 ± 2106.82		944.94 (548.00–1599.25)	
Stage					
0–II	9	7152.91 ± 1663.66	0.045*	558.88 (453.75–758.25)	0.002*
III	12	6993.21 ± 1958.07		727.44 (446.50–1073.63)	
IV	10	8946.22 ± 1913.04		944.94 (548.00–1599.25)	

^a^Presented by mean ± standard deviation

^b^Presented by median values and range

*p < 0.05

Moreover, we chose 10 patients for each of two additional groups. One group consisted of 10 patients with cholesterol polyps who were treated by surgery in the Affiliated Union Hospital of Fujian Medical University. The control group included 10 healthy volunteers. None of the cases had received any preoperative radiochemotherapy or transfusion.

The study was approved by the institutional review board guidelines, according to the Declaration of Helsinki.

### Serum VEGF-C and VEGF-D assay with ELISA

Venous blood samples were collected according to standard hospital procedures before surgery or anticancer therapy. We used serum separators tubes (SST) and allowed the samples to clot for 30 min before centrifugation for 5 min at 2000 × *g*. Separated serum was stored at ≤ −80 °C for future use.

The values of the serum VEGF-C and VEGF-D in the collected samples were determined using the Quantikine human VEGF-C immunoassay kit (R&D Systems, Minneapolis, USA) and the Quantikine human VEGF-D immunoassay kit (R&D Systems, Minneapolis, USA) according to the manufacturers’ instructions, respectively. All samples were assayed in duplicate, and the average was considered the serum level.

The minimum detectable dose (MDD) of VEGF-C ranged from 4.0 to 48.4 pg/ml. The mean MDD was 13.3 pg/ml. The MDD of VEGF-D ranged from 4.7 to 31.3 pg/ml. The mean MDD was 11.4 pg/ml. The coefficient of variation was less than 5.0 %.

### Statistical analysis

The Shapiro-Wilk test was used to examine the normal distribution of the data (VEGF-C *p* = 0.827; VEGF-D *p* = 0.001). Because the sVEGF-C data had a normal distribution, all data from the sVEGF-C groups were presented as the mean ± standard deviation. The differences between these data and the clinicopathological characteristics were evaluated using Student’s *t* test for two groups and a one-way analysis of variance (ANOVA) for more than two groups. However, the sVEGF-D data did not have a normal distribution, and all data from the sVEGF-D groups were characterized by the median and range. The Mann-Whitney *U* test was used to assess the differences between two groups, and the Kruskal-Wallis test was applied to compare three or more groups. The Spearman correlation coefficient test was used to determine the relationship between sVEGF-C and sVEGF-D levels observed in the GBC patients. Kaplan-Meier survival analysis was used to estimate the survival time, and the Mantel’s long-rank test was used to compare the differences in survival time. The COX hazard model was used to explore the independent factors of survival and metastasis status based on the variables selected in the univariate and multivariate analyses. We used the ROC curve to determine the cutoff values of sVEGF-C/D for predicting the presence of disease and LNM. The diagnostic values included the sensitivity, specificity, accuracy, and area under the curve (AUC). Moreover, a parallel method was applied to further predict the presence of disease and LNM. The aforementioned statistical analyses were calculated with the IBM SPSS Statistics 19.0, and the figures were generated in the GraphPad Prism 5 program.

All *p* values were based on a two-tailed statistical analysis. All the results were considered statistically significant at *p* < 0.05.

## Results

### The sVEGF-C/D levels in the patient group

**Fig. 1:**
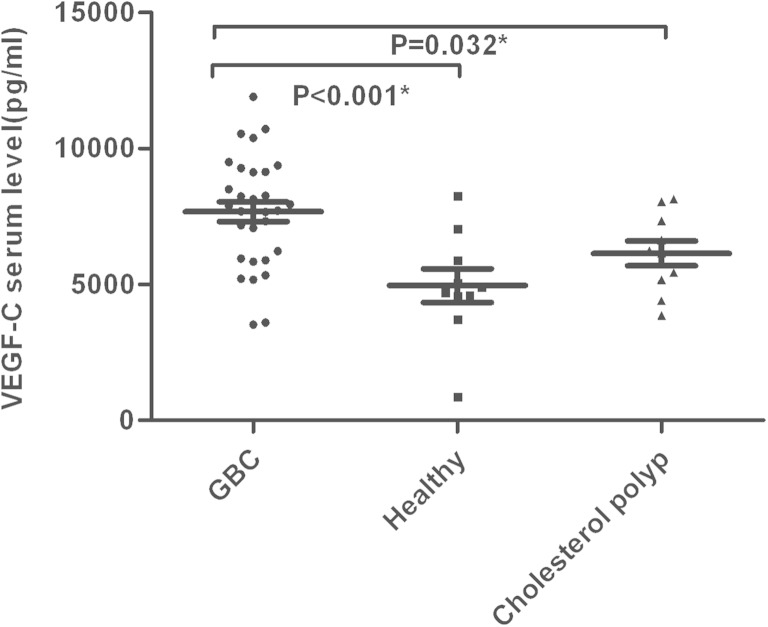
The serum VEGF-C levels are presented in GBC (*n* = 31), healthy volunteers (*n* = 10), and cholesterol polyp (*n* = 10). *p < 0.05

**Fig. 2:**
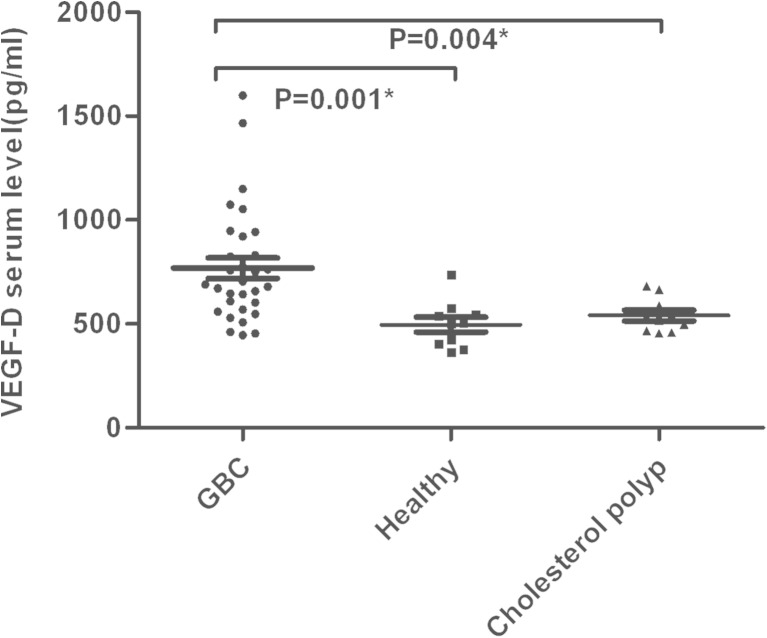
The serum VEGF-D levels are presented in GBC (*n* = 31), healthy volunteers (*n* = 10), and cholesterol polyp (*n* = 10). *p < 0.05

In GBC patients, the mean sVEGF-C levels and median sVEGF-D levels were significantly higher than the healthy group (7669.58 ± 2012.00 vs 4951.55 ± 1963.48 pg/ml; *p* < 0.001 and 689.38 vs 502.69 pg/ml, *p* = 0.001, respectively) and the group with cholesterol polyps of the gallbladder (7669.58 ± 2012.00 vs 6134.51 ± 1449.65 pg/ml, *p* = 0.032 and 689.38 vs 526.25 pg/ml, *p* = 0.004, respectively) (Figs. 1 and 2).

To evaluate the correlation between the sVEGF-C and sVEGF-D levels, we used the Spearman Rank Correlation. A significant and positive correlation was found between sVEGF-C and sVEGF-D (rho = 0.684, *p* < 0.001).

### Association between the clinicopathological feature and the sVEGF-C/D levels of GBC (Table 1)

The sVEGF-C levels were associated with LNM, distant metastasis, and stage (*p* = 0.011, *p* = 0.018 and *p* = 0.045, respectively). However, there was no association with age, sex, smoking status, location, tumor size, histological type, or tumor depth. In GBC patients, the sVEGF-C levels for patients with LNM were significantly higher than those observed for patients without LNM (8477.65 ± 2018.32 vs 6807.64 ± 1663.66 pg/ml, *p* = 0.018). The sVEGF-D levels had a significant association with the tumor depth, LNM, distant metastasis, and stage (*p* = 0.001, *p* = 0.001, *p* = 0.047, and *p* = 0.002, respectively), but it was not associated with age, sex, smoking status, location, tumor size, histological type, or histological grading. In GBC patients, there was a significant difference between the sVEGF-D levels of patients with and without LNM (876.06 vs 602.38 pg/ml, *p* = 0.001).

### ROC curve analysis

To further explore the diagnostic values, we used the following definitions: If one of the sVEGF-C/D levels was higher than the cutoff value, it was identified as positive in the combination (the parallel method). Moreover, all patients underwent an examination of the CA 19-9 and CEA before operation. Elevation of the CA 19-9 level was defined as >37 U/ml, and elevation of the CEA level was defined as >5 ng/ml.

**Fig. 3:**
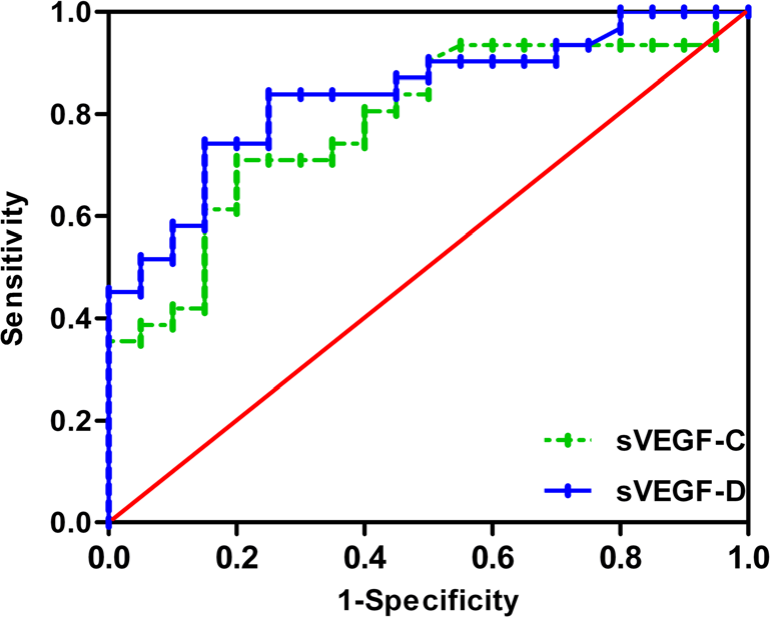
ROC curve analysis for predicting the presence of GBC by serum VEGF-C and VEGF-D (cut-off values 7054.83 and 595.13 pg/ml, respectively)

When the cutoff values of sVEGF-C/D for the diagnosis of GBC patients were 7054.83 and 595.13 pg/ml, the diagnostic values were as follows: sensitivities of 71.0 and 74.2 %, specificities of 80.0 and 85.0 %, accuracies of 74.5 and 78.4 %, and AUCs of 0.785 and 0.838, respectively. There was no significant difference between sVEGF-C and sVEGF-D as biomarkers of GBC (*p* = 0.463) (Fig. 3). When sVEGF-C and sVEGF-D were associated, the sensitivity was 80.6 %, the specificity was 65.0 %, and the accuracy was 74.5 %. The comparison with other markers is shown in Table 2.

**Table 2:** Comparison of VEGF-C/D, CEA, and CA19-9 in predicting the presence of GBC

Parameters	Sensitivity	Specificity	PPV^a^	NPV^b^	Accuracy
VEGF-C	71.0 %	80.0 %	84.6 %	64.0 %	74.5 %
VEGF-D	74.2 %	85.0 %	88.5 %	68.0 %	78.4 %
VEGF-C + D	80.6 %	65.0 %	78.1 %	68.4 %	74.5 %
CEA	41.9 %	95.0 %	92.9 %	51.4 %	62.8 %
CA19-9	54.8 %	90.0 %	89.5 %	56.3 %	68.6 %
CEA + CA19-9	64.5 %	85.0 %	87.0 %	60.7 %	74.0 %

^a^Positive predictive value

^b^Negative predictive value

**Fig. 4:**
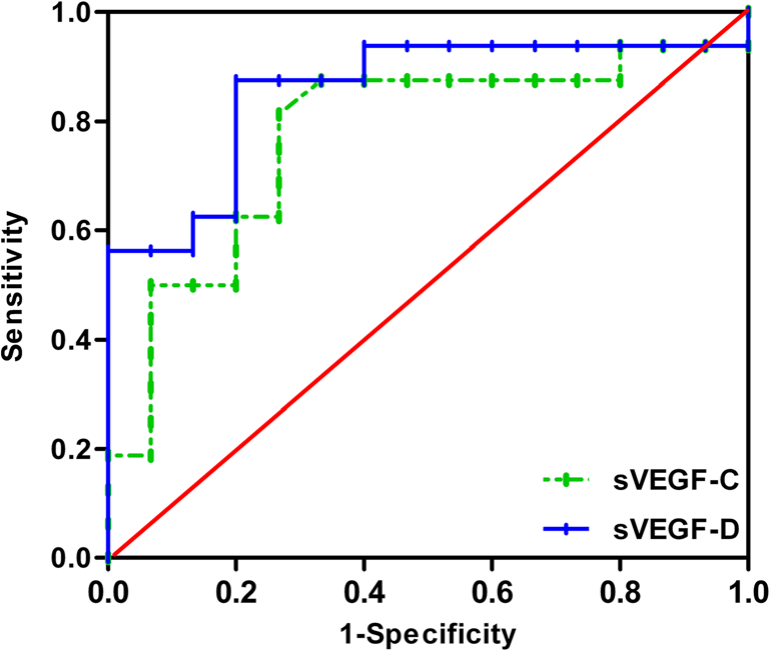
ROC curve analysis for the prediction of LNM by serum VEGF-C and VEGF-D (cut-off values 7667.27 and 674.88 pg/ml, respectively)

With respect to the diagnosis of LNM, when the cutoff values of 7667.27 and 674.88 pg/ml were selected, the diagnostic values of sVEGF-C /D were as follows: sensitivities of 81.2 and 87.5 %, specificities of 73.3 and 80.0 %, accuracies of 77.4 and 83.9 %, and AUCs of 0.773 and 0.854, respectively (Fig. 4). No significant difference was found between sVEGF-C and sVEGF-D as biomarkers of LNM (*p* = 0.28). When we estimated the presence of LNM by combining sVEGF-C with sVEGF-D, this method reached a sensitivity of 87.5 %, a specificity of 60.0 %, and an accuracy of 74.2 %.

### Prognosis analysis

**Fig. 5:**
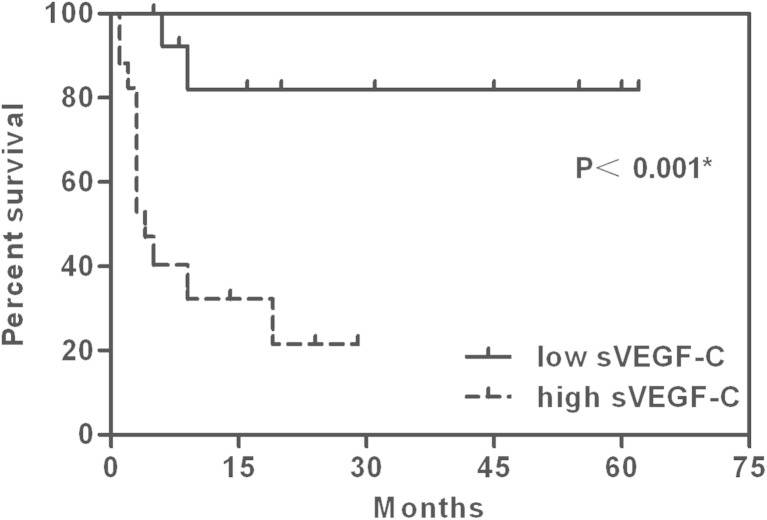
Kaplan-Meier survival curves in the low sVEGF-C (<7669.58 pg/ml) and high sVEGF-C groups (>7669.58 pg/ml). *p < 0.05

**Fig. 6:**
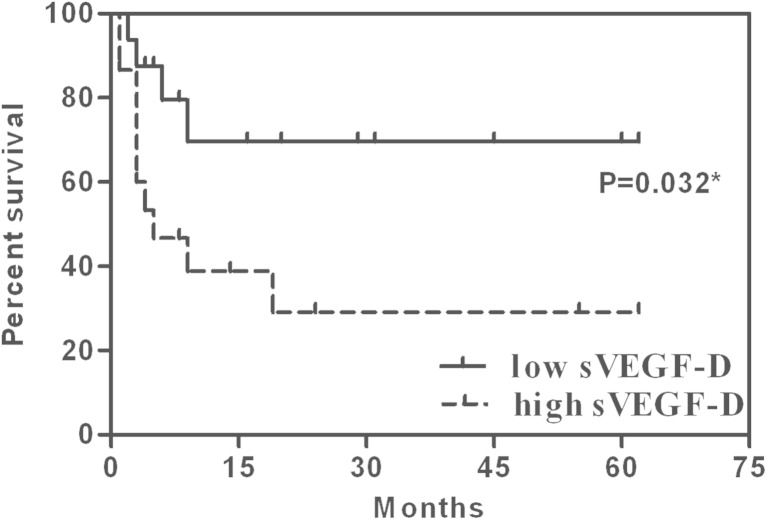
Kaplan-Meier survival curves in the low sVEGF-D (<689.38 pg/ml) and high sVEGF-D groups (>689.38 pg/ml). *p < 0.05

In 31 patients, the median survival time was 19 months, and the median follow-up time was 24 months. The follow-up rate reached 93.5 %. The 1-year survival rate was 63.8 %. The mean survival time of patients with a high sVEGF-C level (>7669.58 pg/ml) was 10.70 months (*n* = 17, 95 % confidence interval (CI) 5.23–16.17), while the low sVEGF-C level (<7669.58 pg/ml) was 52.26 months (*n* = 14, 95 % CI 39.86–64.65) (*p* < 0.001). The high sVEGF-D group (>689.38 pg/ml) had a mean survival time of 22.16 months (*n* = 15, 95 % CI 8.23–36.09), but the low sVEGF-D group (<689.38 pg/ml) had a mean survival time of 44.84 months (*n* = 16.95 % CI 30.39–59.29) (*p* = 0.032) (Figs. 5 and 6).

We performed univariate analysis for the clinicopathological factors and sVEGF-C/D levels that might affect survival and further performed multivariate analysis using the variables that were significant in the univariate analysis. The overall survival rates of GBC patients were independently associated with the TNM stage (*p* = 0.013) and sVEGF-C level (*p* = 0.018) (Table 3).

**Table 3:** Results of COX’s univariate and multivariate analysis

Factors	Univariate analysis			Multivariate analysis		
	Odd ratio	95 % CI	*p* value	Odd ratio	95 % CI	*p* value
Age	0.976	0.918–1.037	0.432			
Sex (male/female)	0.531	0.182–1.548	0.246			
Smoking Status (smoker/non-smoker)	0.463	0.161–0.337	0.155			
Tumor location (neck/body/bottom/whole)	0.864	0.528–1.414	0.561			
Tumor size (cm)	1.370	0.470–3.992	0.564			
Histological type (adeno/others)	1.045	0.583–1.874	0.882			
Histological grading (poor/moderate/well differentiation)	0.498	0.210–1.182	0.114			
Tumor depth (Tis-T2/T3/T4)	4.203	1.837–9.616	0.001*	0.349	0.053–2.294	0.273
LNM (N0/N1/N2)	4.051	1.83–8.965	0.001*	0.277	0.044–1.725	0.169
Distant metastasis (M0/M1)	8.795	2.754–28.087	<0.001*	1.548	0.214–11.219	0.665
Stage (0–II/III/IV)	6.844	2.408–19.449	<0.001*	68.659	2.466–1191.634	0.013*
sVEGF-C (pg/ml)	0.122	0.027–0.551	0.006*	0.006	0–0.412	0.018*
sVEGF-D (pg/ml)	0.312	0.098–0.998	0.05*	2.171	0.38–12.413	0.384

*p < 0.05

## Discussion

We demonstrated that the sVEGF-C and sVEGF-D levels were significantly higher in GBC patients than in healthy people and patients with cholesterol polyps of the gallbladder. Additionally, the sVEGF-C and sVEGF-D levels in GBC patients had a significant, positive correlation. These findings suggested that the sVEGF-C and sVEGF-D levels were lower in the noncancerous gallbladder when transitioning into cancerous cells, and the tumor cells would secrete high levels of VEGF C/D, thereby inducing the growth of tumor lymphatic vessels. Our finding was in accordance with the studies of lung cancer [13] and esophageal cancer [14]. These studies reported that the sVEGF-C and sVEGF-D levels in the patients with carcinoma were significantly higher than the levels observed in healthy people or those with benign disease. However, several studies in gastric cancer patients have reported conflicting results. Tsirlis et al. [15] from Greece observed that the preoperative sVEGF-C level was significantly lower than that observed in controls but that the preoperative sVEGF-D level was significantly higher than that in controls. In contrast, Al-Moundhri et al. [16] from Oman demonstrated that there were no significant differences in the serum VEGF-C levels between gastric adenocarcinoma patients and controls, whereas the serum levels of VEGF-D were significantly higher in control subjects than in gastric adenocarcinoma patients. These results suggested that different cancers and areas might lead to different sVEGF-C and sVEGF-D expression. VEGF-C and VEGF-D can both combine with VEGFR-2 and VEGFR-3, and they might compete with each other [17]. Therefore, the expression depends on respective binding to receptors, which may be influenced by many factors in the cohort of patients, such as the tumor type, area, and advanced tumor stage presentation [16]. There seemed to be no significant differences in the binding between VEGF-C and VEGF-D in our study. In contrast, VEGF-C and VEGF-D cooperate in the occurrence and progression of GBC. More studies are required to validate these assertions.

This study has analyzed the associations between the clinicopathological characteristics and the sVEGF-C/D levels in GBC. We found that the levels of sVEGF-C were related to the LNM, distant metastasis, and clinical stage. Additionally, there were significant correlations between the levels of sVEGF-D and tumor depth, LNM, distant metastasis, and clinical stage. We demonstrated that sVEGF-C/D was upregulated in advanced GBC. The sVEGF-C/D levels in patients with LNM were significantly higher than those in patients without LNM. A gradual increasing trend was observed in the levels of sVEGF-C/D from N0 to N2. Tumor cells could increase the secretion of VEGF-C/D, which could induce lymphatic vascular growth and increase the risk of metastasis. Our observations were consistent with those of some other studies. Nakashima et al. [18] suggested that VEGF-C expression was significantly correlated with lymph node metastasis in GBC patients. A study on papillary thyroid carcinoma [11] found that the serum VEGF-D levels in patients with LNM were significantly higher than in those without metastases. Additionally, VEGF-D overexpression has previously been observed in gastric cancer patients with distant metastasis [19]. Moreover, another study on esophageal cancer [14] reported that the upregulation of the sVEGF-C levels correlated with LNM, the stage, and distant metastases and that elevated levels of sVEGF-D were associated with the tumor depth, stage, and LNM. However, the sVEGF-C levels do not correlate with LNM in the cervical squamous cell carcinoma [20]. Some studies [14, 20] have reported that VEGF-C overexpression has a significant correlation with tumor size, but we did not find an association between them. It seems that there is no association between the tumor size and stage but that the stage is associated with the VEGF-C/D levels. Therefore, we inferred that there is no significant correlation between the tumor size and VEGF-C/D levels.

It has been suggested that the serum VEGF-C/D might predict the presence of malignancy. Previous results [14] have demonstrated that the diagnostic values of the serum VEGF-C/D levels, when used as markers of esophageal cancer, were as follows: sensitivities of 60 and 52 % and specificities of 80 and 78 %. In our study, when the sVEGF-C/D levels reached the cutoff values of 7054.83 and 595.13 pg/ml, the diagnosis values for the presence of malignancy were sensitivities of 71 and 74.2 % and specificities of 80.0 and 85.0 %, but no significant difference was found between sVEGF-C and sVEGF-D when they were used as biomarkers of GBC. At present, other markers such as the serum CA242, CEA, and CA 19-9 levels are often used to predict the presence of GBC in clinical practice [21, 22]. This study compares the differences in the VEGF-C/D, CEA, and CA 19-9 serum levels, both independently and in combination, as diagnostic markers for GBC. We found that the sensitivities of the serum CEA and CA 19-9 levels are too low, which can more easily result in misdiagnosis. According to our study, the serum VEGF-C/D levels may be more promising tumor markers for GBC than CEA and CA 19-9, particularly in terms of sensitivity.

It has been confirmed that the circulating VEGF-C/D levels can distinguish between the presence and absence of LNM in various malignancies. In the gastric cancer, the level of SVEGF-C reached the highest sensitivity (82.8 %) and specificity (81.8 %) for diagnosing LNM when a cutoff value of 542.5 ng/L was used [10]. Additionally, in colorectal cancer, the predicted LNM sensitivity and specificity of SVEGF-C were 85.7 and 80.0 %, respectively (875 pg/ml cutoff) [23]. When using the cutoff point of 215.04 pg/ml in papillary thyroid carcinoma, the corresponding sensitivity and specificity for serum VEGF-D were 69.6 and 62.7 %, respectively [11]. The diagnostic values of sVEGF-C and sVEGF-D as lymph node markers in esophageal cancer were as follows: sensitivities of 74 and 58 % and specificities of 61 and 50 % [14]. In our study, when the cutoff values of the sVEGF-C/D levels as the markers of LNM were 7667.27 and 674.88 pg/ml, respectively, the predicted values were as follows: sensitivities of 81.2 and 87.5 % and specificities of 73.3 and 80 %. Therefore, we suggested that the serum VEGF-C/D levels might be the reliable biomarkers for LNM in GBC.

To further evaluate the values, we predicted the presence of disease and LNM using a combination of the sVEGF-C and sVEGF-D levels. Through this test, the sensitivity was higher than single-marker assays, but lower accuracy was obtained. It is generally known that improving the sensitivity can reduce the rate of missed diagnosis, which is of great importance for GBC, whose diagnosis is often missed. Our findings were in agreement with the study on non-small cell lung cancer [24], which showed that by using the combination of high sVEGF and sVEGF-C levels, 84.2 % of the cases had positive lymph nodes, which was a significantly higher rate than found when using single-marker assays.

In this study, we observed that the mean survival time with high sVEGF-C was significantly shorter than that observed with low sVEGF-C, as well as high sVEGF-D. The COX analysis showed that the sVEGF-C level might be an independent risk factor of the outcome for GBC. These findings were consistent with those reports showing that a high sVEGF-C level was related to poor prognosis in patients with breast tumors [25] and gastric cancer [10]. Other studies have reported that overexpression of VEGF-C was correlated with a poor outcome in GBC patients [18] and that the serum VEGF-D level in PTC patients might be a useful marker for predicting the outcome [11]. These studies suggested that sVEGF-C/D could be useful in predicting the prognosis of GBC patients, which could be because high sVEGF-C/D levels in GBC patients increase the risk of LNM, distant metastasis, and advanced stage, thereby decreasing the chance of radical resection and resulting in poor outcomes. However, colorectal cancer patients with low sVEGF-C levels showed a poorer overall survival than did those with high sVEGF-C levels [26]. In esophageal cancer, there is a significant correlation between the pretreatment serum levels of VEGF-C and survival in patients undergoing surgery, instead of serum VEGF-D [14]. Therefore, further experiments are required to rigorously test these conjectures.

To the best of our knowledge, our study is the first to describe the serum VEGF-C/D levels in patients with GBC and their values in the diagnosis and prognosis. However, the study has some limitations that require further discussion. First, because of the small sample size of GBC patients, which resulted in wide confidence intervals in our analysis, the findings could easily be spurious; thus, larger sample sizes will be needed in future studies. Second, the postoperative serum VEGF-C/D levels should be measured and compared with the preoperative levels to compare the diagnosis with sVEGF-C/D. Another limitation was the possibility of selection bias because not all GBC patients could provide serum for the analysis, and we finally settled on a sample size of 31 GBC patients.

In conclusion, our work suggested that the sVEGF-C/D levels could be used as reliable biomarkers for predicting the metastasis status and prognosis of GBC patients, thus enabling preparation for and selection of the best treatment in advance.
